# Draft Genome Sequence of *Lactobacillus salivarius* SGL 03, a Novel Potential Probiotic Strain

**DOI:** 10.1128/genomeA.01340-17

**Published:** 2017-12-07

**Authors:** Federica Federici, Laura Manna, Eleonora Rizzi, Elena Galantini, Umberto Marini

**Affiliations:** Sintal Dietetics s.r.l., Castelnuovo Vomano, Teramo, Italy

## Abstract

In this work, we report the draft genome sequence of *Lactobacillus salivarius* SGL 03, a novel potential probiotic strain isolated from healthy infant stools. Antibiotic resistance analysis revealed the presence of a tetracycline resistance gene without elements potentially responsible for interspecific horizontal gene transfer.

## GENOME ANNOUNCEMENT

*Lactobacillus salivarius* is commonly found in the oral cavity and gastrointestinal tract of humans and other mammals. Nowadays, the *L. salivarius* clade comprises 12 species that have been isolated from human, animal, food, and environmental samples. Several potential host-advantageous properties have been attributed to many strains, making this species of interest for probiotic use in the human and veterinary fields (1, 2). Here, we report the draft genome sequence of *Lactobacillus salivarius* SGL 03, isolated from healthy infant stools.

Strain SGL 03 was cultivated in de Man-Rogosa-Sharpe (MRS) broth at 37°C for 24 h before biobanking and DNA extraction. Genomic DNA was purified with the Wizard genomic DNA purification kit (Promega), according to the manufacturer’s instructions. For genome sequencing, a paired-end library (Nextera XT) was sequenced with a HiSeq 2500 instrument (Illumina), providing high-fidelity short reads. This sequencing approach was coupled by sequencing a 6-kb genomic library in a single-molecule real-time (SMRT) cell of the PacBio RSII platform (Pacific Biosciences), leading to long reads used as an assembly scaffold. Illumina reads were filtered for quality standards using CASAVA 1.8.3; PacBio reads were filtered with BBMap 34.46 and assembled using ABySS 1.5.1 in order to create contigs where Illumina reads were aligned using BLASR (3); this made superscaffolds whose order and orientation were checked with SSPACE-LongRead scaffolder 1.0 (4, 5). Missing regions in the superscaffolds were filled using the GapFiller 1.10 software (6). The genome was assembled into 5 final contigs for a total of 1,989,039 bp.

The genome was annotated using the Prokka software (7), with default parameters, obtaining the outputs in GenBank format. The *L. salivarius* SGL 03 draft genome consists of 5 scaffolds, and its total length is 1,989,039 bp, with a coverage of 718× and a G+C content of 41.9%. The largest contig obtained is 175,092 bp. The total *L. salivarius* SGL 03 draft genome contains 2,335 coding sequences, of which 1,964 encode proteins and 641 encode hypothetical proteins. A comparison of SGL 03 with *L. salivarius* JCM 1046 revealed the presence of a megaplasmid of approximately 0.17 Mb and 33.15% G+C content.

Antibiotic resistance characterization was performed using the BLAST (8) software against the ARDB database (9), showing a putative tetracycline resistance gene (*tetA*). The gene has no elements potentially responsible for interspecific horizontal gene transfer in syntenic regions (±500 bp from start and stop codons) (10). The annotated shotgun sequence was exported in GenBank format and deposited on NCBI’s whole-genome sequencing (WGS) portal.

### Accession number(s).

This whole-genome shotgun project has been deposited at DDBJ/ENA/GenBank under the accession number PECX00000000. The version described in this paper is version PECX01000000.
